# RpS13 controls the homeostasis of germline stem cell niche through Rho1‐mediated signals in the *Drosophila* testis

**DOI:** 10.1111/cpr.12899

**Published:** 2020-09-08

**Authors:** Min Wang, Xia Chen, Yibo Wu, Qianwen Zheng, Wanyin Chen, Yidan Yan, Xiaojin Luan, Cong Shen, Jie Fang, Bo Zheng, Jun Yu

**Affiliations:** ^1^ Department of Gynecology the Affiliated Hospital of Jiangsu University Jiangsu University Zhenjiang China; ^2^ Human Reproductive and Genetic Center Affiliated Hospital of Jiangnan University Wuxi, Jiangsu China; ^3^ State Key Laboratory of Reproductive Medicine Center for Reproduction and Genetics Suzhou Municipal Hospital the Affiliated Suzhou Hospital of Nanjing Medical University Suzhou China

**Keywords:** differentiation, germline stem cell, Rho1, RpS13, self‐renewal, stem cell niche

## Abstract

**Objectives:**

Stem cell niche regulated the renewal and differentiation of germline stem cells (GSCs) in *Drosophila*. Previously, we and others identified a series of genes encoding ribosomal proteins that may contribute to the self‐renewal and differentiation of GSCs. However, the mechanisms that maintain and differentiate GSCs in their niches were not well understood.

**Materials and Methods:**

Flies were used to generate tissue‐specific gene knockdown. Small interfering RNAs were used to knockdown genes in S2 cells. qRT‐PCR was used to examine the relative mRNA expression level. TUNEL staining or flow cytometry assays were used to detect cell survival. Immunofluorescence was used to determine protein localization and expression pattern.

**Results:**

Herein, using a genetic manipulation approach, we investigated the role of ribosomal protein S13 (RpS13) in testes and S2 cells. We reported that RpS13 was required for the self‐renewal and differentiation of GSCs. We also demonstrated that RpS13 regulated cell proliferation and apoptosis. Mechanistically, we showed that RpS13 regulated the expression of ribosome subunits and could moderate the expression of the Rho1, DE‐cad and Arm proteins. Notably, Rho1 imitated the phenotype of RpS13 in both *Drosophila* testes and S2 cells, and recruited cell adhesions, which was mediated by the DE‐cad and Arm proteins.

**Conclusion:**

These findings uncover a novel mechanism of RpS13 that mediates Rho1 signals in the stem cell niche of the *Drosophila* testis.

## Graphical abstract

Objectives: Stem cell niche regulated the renewal and differentiation of germline stem cells (GSCs) in *Drosophila*. Previously, we and others identified a series of genes encoding ribosomal proteins that may contribute to the self‐renewal and differentiation of GSCs. However, the mechanisms that maintain and differentiate GSCs in their niches were not well understood.Materials and Methods: Flies were used to generate tissue‐specific gene knockdown. Small interfering RNAs were used to knockdown genes in S2 cells. qRT‐PCR was used to examine the relative mRNA expression level. TUNEL staining or flow cytometry assays were used to detect cell survival. Immunofluorescence was used to determine protein localization and expression pattern.Results: Herein, using a genetic manipulation approach, we investigated the role of ribosomal protein S13 (RpS13) in testes and S2 cells. We reported that RpS13 was required for the self‐renewal and differentiation of GSCs. We also demonstrated that RpS13 regulated cell proliferation and apoptosis. Mechanistically, we showed that RpS13 regulated the expression of ribosome subunits and could moderate the expression of the Rho1, DE‐cad and Arm proteins. Notably, Rho1 imitated the phenotype of RpS13 in both *Drosophila* testes and S2 cells, and recruited cell adhesions, which was mediated by the DE‐cad and Arm proteins.Conclusions: These findings uncover a novel mechanism of RpS13 that mediates Rho1 signals in the stem cell niche of the *Drosophila* testis.

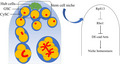

## 1 INTRODUCTION

The stem cell niche in *Drosophila* provides a special microenvironment through microenvironment cells and specific extracellular matrix and adhesion molecules. It plays key roles in maintaining a high proliferation rate and inducing directional differentiation of stem cells, as well as a strict balance of stem cells during stem cell homeostasis.^1^, ^2^ Previously, *Drosophila* was identified as a potential model organism for understanding stem cells and niche signals.^3^

At the apex of the *Drosophila* testis, approximately ten to fifteen non‐mitotic somatic support cells (called the hub cells) produce sophisticated and well‐ordered signals that directly interact with two stem cell populations: germline stem cells (GSCs) and somatic cyst stem cells (CySCs), which are strictly controlled by local signals.^3^ For instance, hub cells secrete Upd protein, which activates the JAK‐STAT signalling pathway in adjacent GSCs and CySCs and is essential to maintain both GSCs and CySCs in the *Drosophila* testis.^4^ Activation of JAK‐STAT signals in CySCs autonomously regulates their self‐renewal and maintenance via the transcriptional repressors Zfh‐1 and Chinmo and other factors.^4^, ^5^ In contrast, GSC characteristics can also be maintained by activating BMP signals from CySCs and hub cells.^6^ Early germ cells (eg GSCs) that move away from hub cells leads to differentiation defects without CySCs, indicating the importance of the instructive differentiation signals from CySCs.^7^ Through internal and external regulatory factors, niche cells (hub cells, GSCs and CySCs) mutually coordinate to control the homeostasis of GSCs.

The classical Wnt/β‐catenin (β‐catenin is also called Arm in *Drosophila*) pathway primarily regulates cell fate determination during development.^8^ Shifting the Arm protein from the cadherin bound pool to the cytoplasmic pool can increase the amount of available Arm protein for the activation of downstream target genes.^9^ As a multifunctional protein, Arm can interact with cadherin in cell adhesions to form a link with the actin cytoskeleton.^9^, ^10^ Moreover, the non‐classical Wnt signalling pathway mainly regulates cytoskeletal organization and has been classified as two independent pathways (Wnt/JNK and Wnt/calcium) depending on their major intracellular mediators.^8^, ^11^ The small GTPases of the Rho family, including Rac1, Cdc42 and Rho1, are involved in the non‐classical Wnt signalling pathway. Rho GTPases act as a molecular switch participating in several physiological activities such as cell migration, cell adhesion, cytokinesis, cell proliferation, apoptosis and differentiation.^12^, ^13^

Up to now, several studies have indicated that GSC self‐renewal and differentiation are controlled by the interplay of several signalling pathways that provide positional information and induce cell fate specification.^5^, ^14^, ^15^ Wnt/β‐catenin signalling has been identified as having key roles in controlling ribosome biogenesis through a Wnt/Myc/Ribosome axis, a key driver of cell proliferation in the mouse.^16^ Significantly, ribosome biogenesis plays key roles in stem cell‐specific mechanisms among stem cells and differentiated cells.^17^

Translation of mitochondrial encoded ribosomal RNAs (mtrRNAs) is required for embryonic germline formation in *Drosophila*.^18^ The ribosome‐specific MrpL55 protein is localized to the mitochondria in *Drosophila* S2 cells.^19^ In the *Drosophila* ovary, ribosomal assembly factors are also important for the regulation of stem cell cytokinesis, and they contribute to the transition from self‐renewal to differentiation.^20^

Previously, we performed a large scale RNAi screen and identified a series of ribosomal proteins involved in GSC maintenance in the *Drosophila* testis, and importantly, RpS13 was one of them.^21^ However, the specific mechanism of RpS13 in the stem cell niche remains unclear. Here, we will systematically analyse the function of RpS13 in GSC self‐renewal and differentiation and explored its regulatory mechanisms on the homeostasis of the stem cell niche in the *Drosophila* testis.

## 2 MATERIALS AND METHODS

### 2.1 Stocks and cross strategy

All flies were maintained on standard *Drosophila* media at 25ºC. The transgenic RNA interference (RNAi) flies used in the study were obtained from TsingHua Fly Center (THFC, Beijing, China). Fly stocks used in this study are described either in FlyBase or as noted: nos‐Gal4 (#4937; Bloomington *Drosophila* Stock Center, Bloomington, IN, USA), tj‐Gal4 (#104055; *Drosophila* Genetic Resource Consortium, Kyoto, Japan), UAS‐RpS13 RNAi (THU0667, THFC) and UAS‐Rho1 RNAi (THU3565, THFC).

UAS‐RNAi virgins were selected to cross with male Gal4 lines and raised at room temperature (25°C), and then, the hatched male offspring of certain genotypes were selected within two days for further experiments.

### 2.2 Cell culture of S2 cells and transfection

*Drosophila* Schneider 2 (S2) cells, obtained from the *Drosophila* Genomics Resource Center, were cultured in Schneider's *Drosophila* medium (21720024, Gibco, USA) supplemented with 10% heat‐inactivated foetal bovine serum (04‐001‐1ACS; Biological Industries, Israel) at 28°C. S2 cells were separated at a ratio of 1:4 every 3‐4 days and replated in the supplemented medium. S2 cells were seeded on 6‐well plates and grown until 70%–80% confluence. For the knockdown assay, S2 cells were transfected using Lipofectamine 2000 (Lipo2000; 11668019, Invitrogen, USA). The siRNAs were designed and synthesized by GenePharma (Suzhou, China), and their detailed information is listed in Table S1.

### 2.3 Quantitative reverse transcription‐polymerase chain reaction (qRT‐PCR)

TRIzol reagent (9108, Takara, Japan) was used to extract total RNA, and then, we used a Prime Script RT Reagent Kit (RR037A, Takara, Japan) to perform reverse transcription. The Agilent Mx3000P Real‐Time PCR System (Agilent Technologies, Santa Clara, CA, USA) was used to perform qRT‐PCR with TB Green^TM^ Premix Ex Taq^TM^ (RR420A, Takara, Japan), and the 2^‐△△Ct^ method (Ct values are threshold cycles) was performed to calculate the relative mRNA levels. All samples were normalized to glyceraldehyde‐3‐phosphate dehydrogenase (GAPDH), which was used as an internal reference gene. Experiments were independently repeated at least three times. Primers used in the qRT‐PCR assay are listed in Table S2.

### 2.4 Immunofluorescence

Immunofluorescence assays were carried out as described previously.^22^ Testes were dissected in 1 × PBS and fixed for 30 minutes in 4% paraformaldehyde (PFA), then washed 3 times with 0.3% PBS‐Triton X‐100 (PBST) and blocked for 1 hour in 5% bovine serum albumin (BSA) (Sangon Biotech, Shanghai, China). Next, the testes were incubated overnight at 4°C with primary antibodies (Table S3) diluted in 5% BSA. After washing 3 times with 0.3% PBST, the testes were incubated with the secondary antibodies for 1 hour at room temperature. The secondary antibodies conjugated to A488 or Cy3 (Molecular Probes and Jackson Immunologicals) were diluted at 1:1000. Finally, the testes were washed 3 times, incubated with Hoechst‐33342 (C0031, Solarbio) at 1.0 mg/mL for 5 minutes and mounted in glycerol solution. Confocal images were obtained with the Zeiss LSM800 system (Carl Zeiss, Oberkochen, Germany) and were processed with Adobe Photoshop Software (Adobe, San Jose, CA, USA). A similar staining process was used for S2 cells.

### 2.5 Detection of apoptosis

The TUNEL BrightRed Apoptosis Detection Kit (Vazyme A113; Nanjing, China) was used for the detection of apoptosis according to the manufacturer's instructions. Cover glasses were put into a 24‐well plate. Then, they were washed 3 times for 5 minutes in 1 × PBS and incubated with poly‐L‐lysine for 2 hours at 28°C. After removing the poly‐L‐lysine, the cover glasses were washed 3 times for 5 minutes again. After transfection for 24 hours, S2 cells were transferred to the 24‐well plate for the cell climbing slice and allowed to grow on the treated cover glasses. After incubation for 24 hours at 28°C, the S2 cells had covered the glass slides, and they were fixed for 20 minutes in 4% PFA, washed 3 times with 0.3% PBST, and were blocked for 30 minutes in 5% BSA. Each sample was balanced with 5 × Equilibration Buffer for 30 minutes at room temperature. BrightRed and TdT enzyme were used to label apoptotic cells for 1 hour at 37°C. After washing 3 times with 0.3% PBST, the samples were incubated with Hoechst‐33342 at 1.0 mg/mL for 15 minutes in the dark, then were washed 3 times and were mounted in glycerol solution.

### 2.6 Flow cytometry assay

FACScan flow cytometry (BD Biosciences, San Jose, CA, USA) was used to detect the ratio of cell death according to the Annexin V‐Alexa Fluor 647/propidium iodide Apoptosis Assay Kit (FMSAV647‐100; FcMACS, Nanjing, China). After transfection for 48 hours, S2 cells (1 × 10^6^ cells/well) from each sample (≥3) were centrifuged at 300 g for 5 minutes, and then, we discarded the supernatant. After suspension of the precipitated cells in 250 µL binding buffer, 10 µL of propidium iodide and 5 µL of Annexin V‐Alexa Fluor 647 were mixed and incubated with the cell suspension for 15 minutes at room temperature in the dark. Before testing on FACScan flow cytometry, 200 µL 1 × PBS was added to each sample to dilute the cells. The experiments were performed at least 3 times.

### 2.7 Statistical analysis

Cell count, distance measurement, fluorescence intensity and expressed area were performed with ImageJ Software (imagej.nih.gov/ij). Experiments were repeated at least 3 times. The quantitative data are presented as the mean ± standard error of the mean (SEM), and the data were evaluated for significant differences using Student's t test and one‐way ANOVA by GraphPad Software (La Jolla, CA, USA). *P* value < .05 was considered indicative of statistical significance.

## 3 RESULTS

### 3.1 RpS13 regulates the function of the stem cell niche in *Drosophila* testes

**Figure 1:**
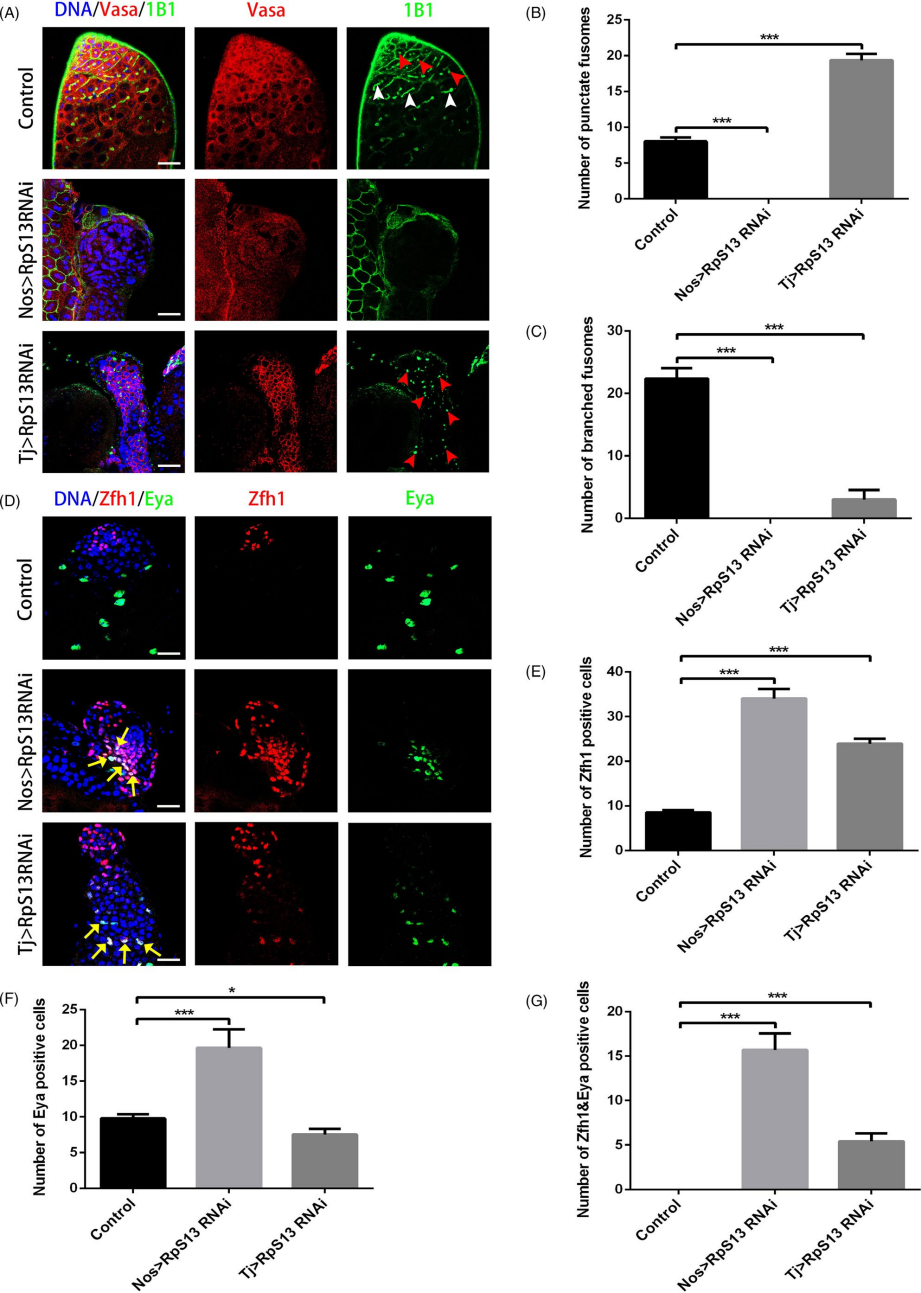
Functional analysis of RpS13 for GSC self‐renewal and differentiation in *Drosophila* testes. A, Apical tips of control, nos > RpS13 RNAi and tj > RpS13 RNAi testes labelled with Vasa (red) and 1B1 (green). Red arrowheads indicate representative punctate fusomes and white arrowheads indicate representative branched fusomes. B, The number of punctate fusomes in the control, nos > RpS13 RNAi and tj > RpS13 RNAi testes. C, The number of branched fusomes in the control, nos > RpS13 RNAi and tj > RpS13 RNAi testes. D, Apical tips of the control, nos > RpS13 RNAi and tj > RpS13 testes labelled with Zfh1 (red) and Eya (green). Yellow arrows indicate representative Zfh1&Eya‐positive cells. E, The number of Zfh1‐positive cells in the control, nos > RpS13 RNAi and tj > RpS13 RNAi testes. F, The number of Eya‐positive cells in the control, nos > RpS13 RNAi and tj > RpS13 RNAi testes. G, The number of Zfh1&Eya‐positive cells in the control, nos > RpS13 RNAi and tj > RpS13 RNAi testes. DNA was stained with Hoechst 33 342 (blue). *P < .05, ***P < .001. Scale bars, 20 µm

To elucidate the roles of RpS13 in *Drosophila* testes, we knocked down RpS13 in early germ cells with a nos‐Gal4 driver and in cyst cells with a tj‐Gal4 driver. Fusome, a germline‐specific endoplasmic reticulum‐like organelle, has a spherical shape in GSCs and gonialblast daughters and has branched structures in differentiated spermatogonia, which can be labelled by 1B1. The known asymmetric segregation of spectrosome/fusome is vital for stem cell maintenance or GSC daughter specification.^23^ We observed that the Vasa‐labelled germ cells and 1B1‐labelled fusomes were totally lost in the nos > RpS13 RNAi testes (Figure 1A‐C). Zfh1 is a marker that is mainly expressed in CySCs and early cyst cells, and Eya is used to mark mature cyst cells. In the nos > RpS13 RNAi testes, we found that Eya‐positive (Eya+) and Zfh1‐positive (Zfh1+) cyst cells accumulated and even Eya/Zfh1 double positive (Eya+/Zfh1+) cells existed, indicating the formation of abnormal characteristics of cyst stem cells or mature cyst cells (Figure 1D‐F).

In contrast, early stage germ cells accumulated along with significant increases of punctate fusomes and decreased amounts of branched fusomes in the tj > RpS13 RNAi testes when compared with controls (Figure 1A‐C), indicating that the loss of RpS13 in cyst cells caused GSC differentiation defects. We also found that Zfh1 + and Eya+/Zfh1 + cyst cells accumulated in tj > RpS13 RNAi testes (Figure 1D,E,G). In Particular, Eya + cyst cells decreased in tj > RpS13 RNAi testes when compared with controls, and this was mainly because of the increase of early stage germ cells (Figure 1D,F).

Usually, mitotic cells marked by PH3 reside around hub cells. We showed that PH3‐positive (PH3+) cells resided far from the hub cells when knocking down RpS13 in cyst cells with the tj‐Gal4 driver (Figure S1). Taken together, these data suggest that RpS13 is required for the maintenance of GSCs by cell autonomous effects, and it is also essential for the differentiation of GSCs via non‐cell autonomous mechanisms, which contribute to the regulation of the stem cell niche in *Drosophila* testes.

### 3.2 RpS13 controls the homeostasis of proliferation and cell death in S2 cells

**Figure 2:**
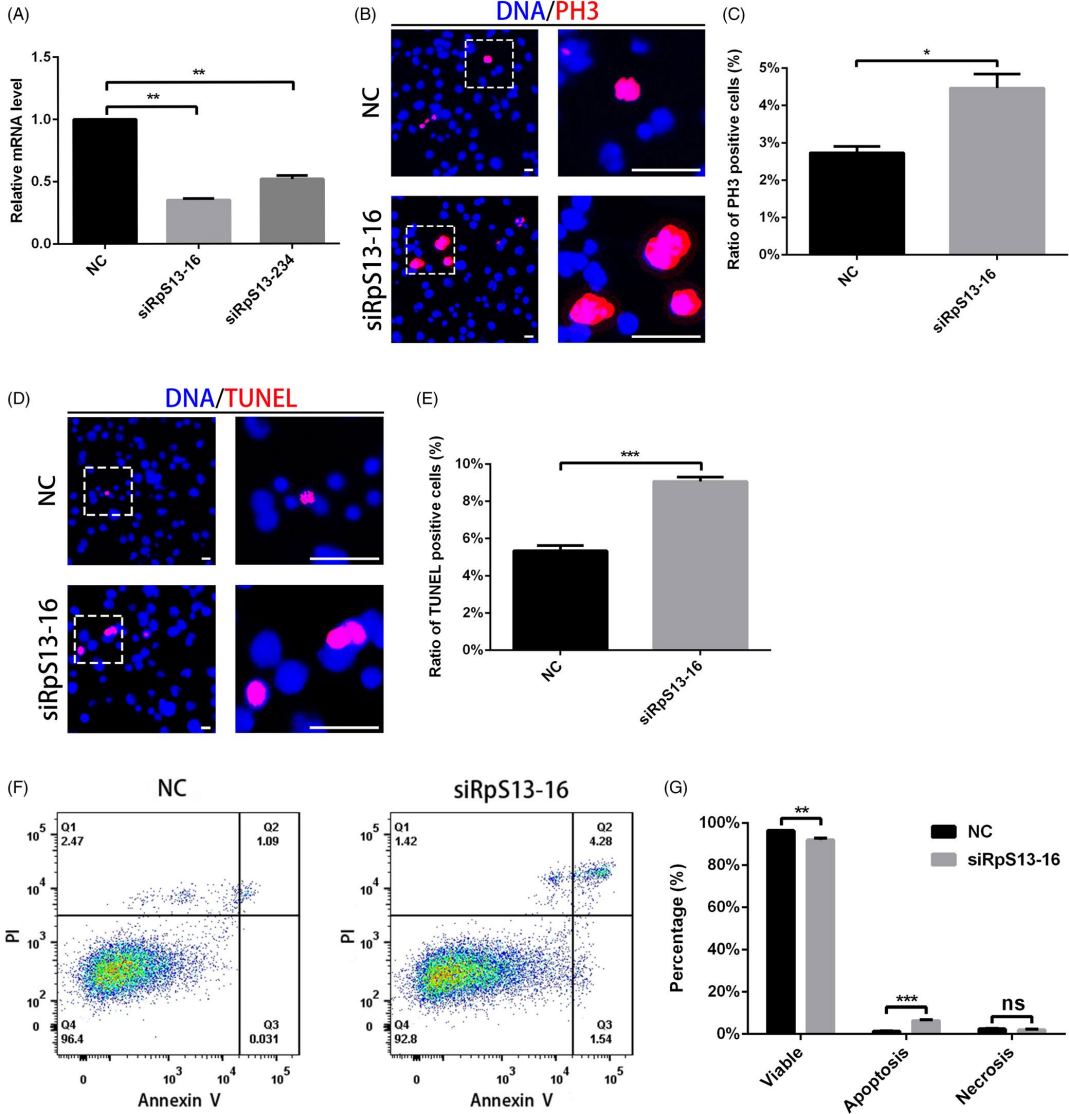
RpS13 controls proliferation and cell death in S2 cells. A, Relative mRNA level of RpS13 in NC and RpS13 siRNA S2 cells. B, Immunostaining of PH3 (red) in NC and RpS13 siRNA S2 cells. C, Ratio of PH3‐positive cells in NC and RpS13 siRNA S2 cells. D, Staining of TUNEL (red) in NC and RpS13 siRNA S2 cells. E, Ratio of TUNEL‐positive cells in NC and RpS13 siRNA S2 cells. F, Flow cytometry test for PI and Annexin V in NC and RpS13 siRNA S2 cells. G, Cell component analysis in NC and RpS13 siRNA S2 cells. *P < .05, **P < .01, ***P < .001, ns represents no significance. Scale bars, 30 μm

The maintenance and differentiation defects of GSCs might be caused by cell fate determination, so next we examined the proliferation and cell death of negative control (NC) and siRpS13‐treated S2 cells. We detected the interference efficiency of two siRNAs (RpS13 siRNA‐16 and RpS13 siRNA‐234) and used RpS13 siRNA‐16 for the following function experiments (Figure 2A). We observed that both PH3 + and TUNEL‐positive (TUNEL+) cells increased in RpS13 siRNA‐16 S2 cells when compared with controls (Figure 2B‐E). Additionally, flow cytometry was used to detect the ratio of apoptosis cells, which was consistent with the results of TUNEL staining (Figure 2F,G). Taken together, these data indicate that RpS13 could regulate proliferation and apoptosis processes in *Drosophila* S2 cells.

### 3.3 Rho1 is required for the homeostasis of the stem cell niche in *Drosophila* testes

**Figure 3:**
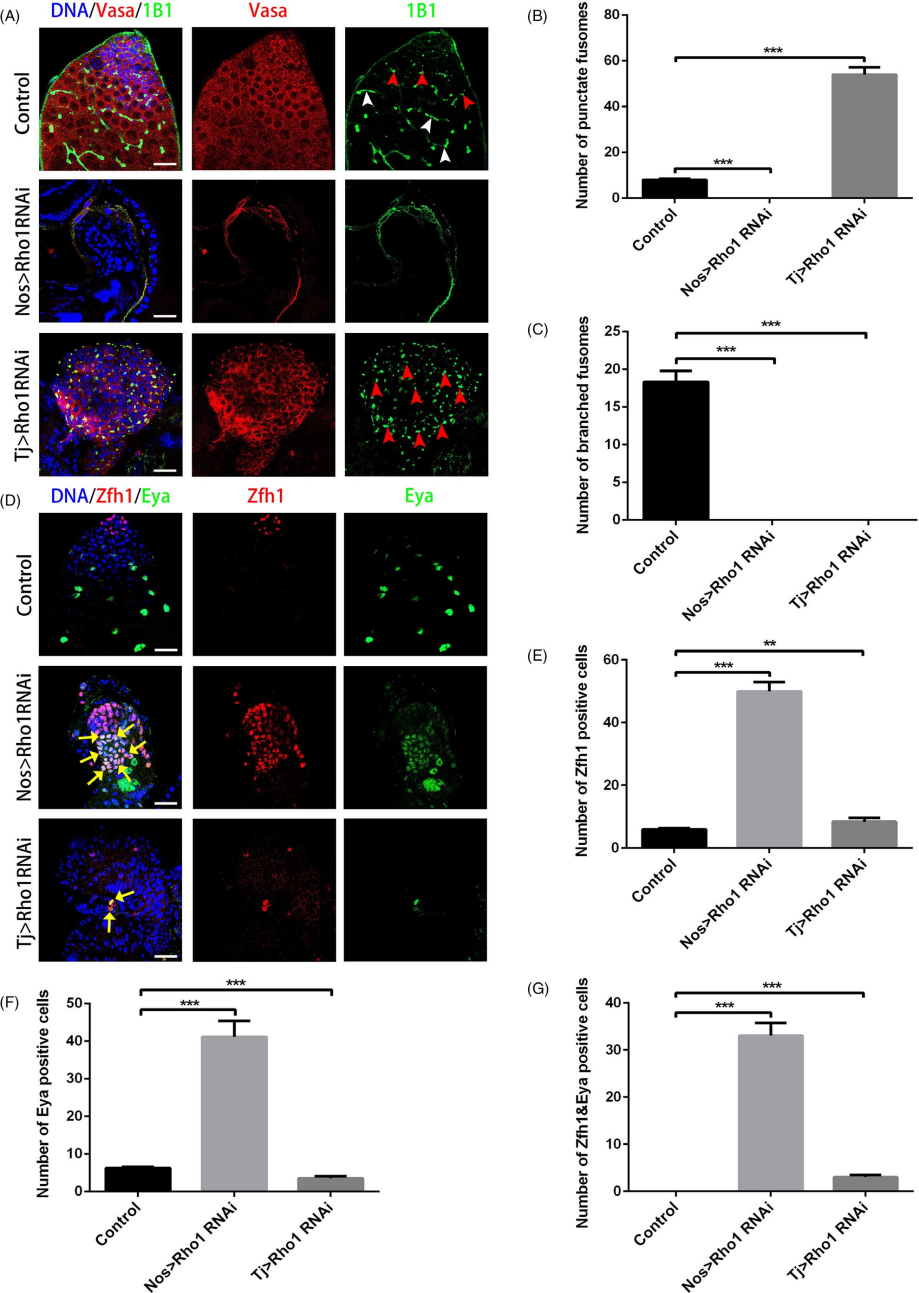
Rho1 regulates the function of the stem cell niche in *Drosophila* testes. A, Apical tips of control, nos > Rho1 RNAi and tj > Rho1 RNAi testes labelled with Vasa (red) and 1B1 (green). Red arrowheads indicate representative punctate fusomes, and white arrowheads indicate representative branched fusomes. B, The number of punctate fusomes in the control, nos > Rho1 RNAi and tj > Rho1 RNAi testes. C, The number of branched fusomes in the control, nos > Rho1 RNAi and tj > Rho1 RNAi testes. D, Apical tips of control, nos > Rho1 RNAi and tj > Rho1 testes labelled with Zfh1 (red) and Eya (green). Yellow arrows indicate representative Zfh1&Eya‐positive cells. E, The number of Zfh1‐positive cells in the control, nos > Rho1 RNAi and tj > Rho1 RNAi testes. F, The number of Eya‐positive cells in the control, nos > Rho1 RNAi and tj > Rho1 RNAi testes. G, The number of Zfh1&Eya‐positive cells in the control, nos > Rho1 RNAi and tj > Rho1 RNAi testes. DNA was stained with Hoechst 33 342 (blue). **P < .01, ***P < .001. Scale bars, 20 µm

Since it was demonstrated that Wnt signalling regulated ribosome biogenesis, we next tested the phenotype of the Rho family GTPases, identified as downstream factors of the non‐classical Wnt signalling pathway. Interestingly, knockdown of Rho1 in early germ cells also resulted in a loss of germ cells and fusomes, and knockdown of Rho1 in cyst cells caused the accumulation of undifferentiated germ cells (Figure 3A‐C). Meanwhile, the number of PH3 + mitotic cells significantly increased, and they resided away from the hub cells (Figure S2), suggesting that these undifferentiated germ cells retained their proliferation ability in the absence of normal hub signals. To further explore the effect of Rho1 on cyst cells, we next stained for Zfh1 and Eya in nos > Rho1 RNAi and tj > Rho1 RNAi testes. Surprisingly, Eya+, Zfh1 + and Eya+/Zfh1 + cyst cells were dramatically accumulated in nos > RpS13 RNAi testes, while only a few cyst cells were scattered in tj > RpS13 RNAi testes (Figure 3D‐G). These results strongly suggest that Rho1 mimicked the phenotype of RpS13 in the stem cell niche of the *Drosophila* testis.

### 3.4 Rho1 regulates proliferation and apoptosis processes in S2 cells

**Figure 4:**
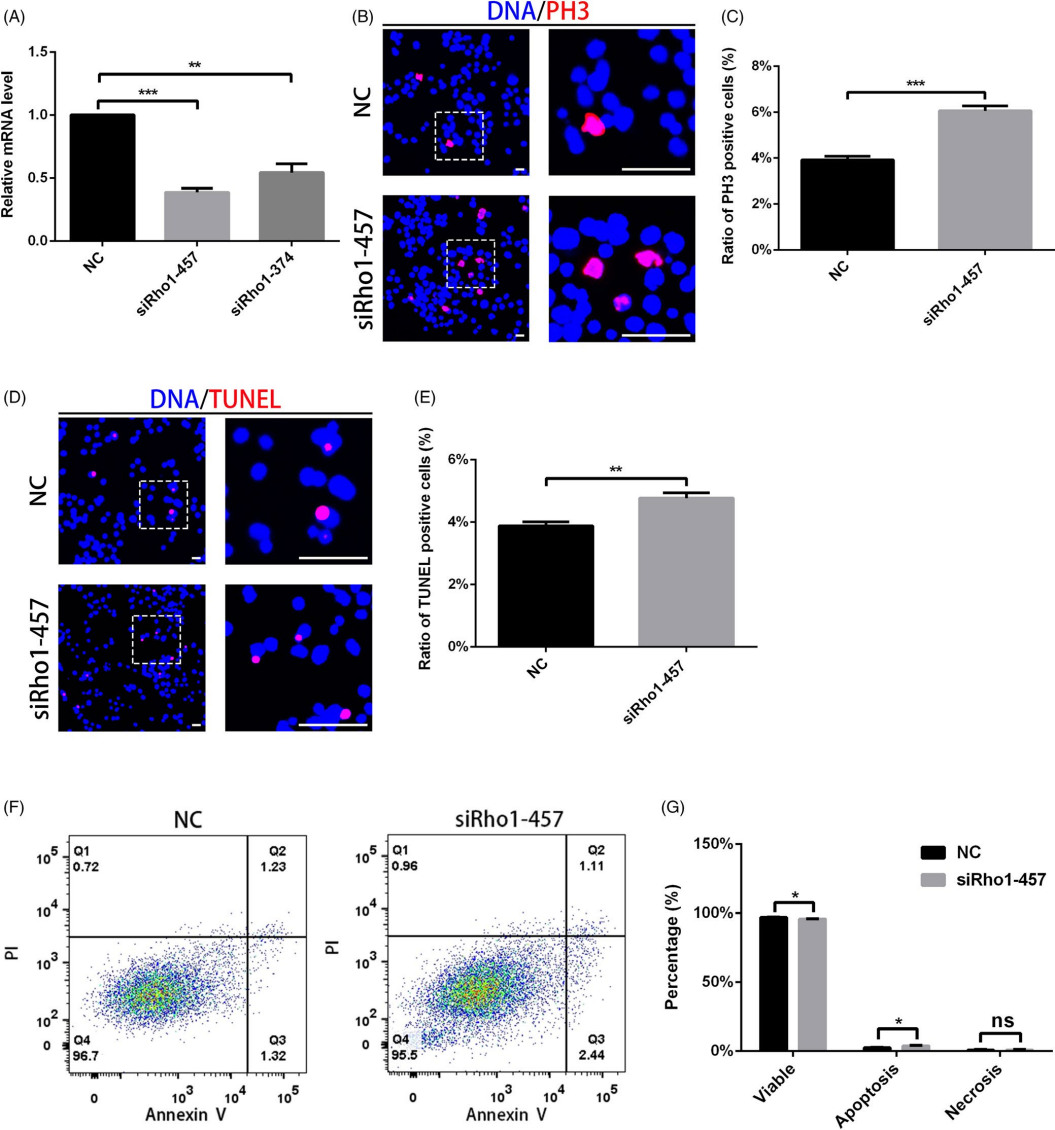
Rho1 regulated proliferation and apoptosis processes in S2 cells. A, Relative mRNA level of Rho1 in NC and Rho1 siRNA S2 cells. B, Immunostaining of PH3 (red) in NC and Rho1 siRNA S2 cells. C, Ratio of PH3‐positive cells in NC and Rho1 siRNA S2 cells. D, Staining of TUNEL (red) in NC and Rho1 siRNA S2 cells. E, Ratio of TUNEL‐positive cells in NC and Rho1 siRNA S2 cells. F, Flow cytometry test for PI and Annexin V in NC and Rho1 siRNA S2 cells. G, Cell component analysis in NC and Rho1 siRNA S2 cells. *P < .05, **P < .01, ***P < .001, ns represents no significance. Scale bars, 30 μm

Rho1 exhibited a similar phenotype to RpS13 in the stem cell niche of *Drosophila* testes, so we further confirmed the effects of Rho1 through downregulation of its expression using siRNA‐mediated Rho1 silencing. We tested the relative expression level of Rho1 siRNA in S2 cells (Figure 4A). Similarly, the staining results showed that both PH3 + and TUNEL + cells increased in Rho1 siRNA S2 cells compared with NC (Figure 4B‐E). Component analysis of cell death by flow cytometry indicated that apoptotic cells increased in Rho1 siRNA S2 cells compared with NC (Figure 4F,G). Taken together, our results indicated that Rho1 regulates both the proliferation and apoptosis processes, which is consistent with the role of RpS13 in S2 cells.

### 3.5 RpS13 regulates Rho1 by moderating DE‐cad‐ and Arm‐mediated cell adhesions

The small GTPase Rho1 was reported to regulate cadherin‐based adherens functions during embryonic morphogenesis and to mediate the mislocalization of DE‐cad protein.^24^, ^25^ The DE‐cadherin‐mediated adherens junction plays roles in the regulation of the stem cell niche in *Drosophila*.^26^ Meanwhile, several studies indicated that Arm could bind to cadherin at adherens junctions and that it was highly expressed in the hub‐GSC interface as well as the hub‐hub interface, showing the significant roles of Arm‐mediated cell adhesions in the stem cell niche.^4^, ^27^, ^28^

**Figure 5:**
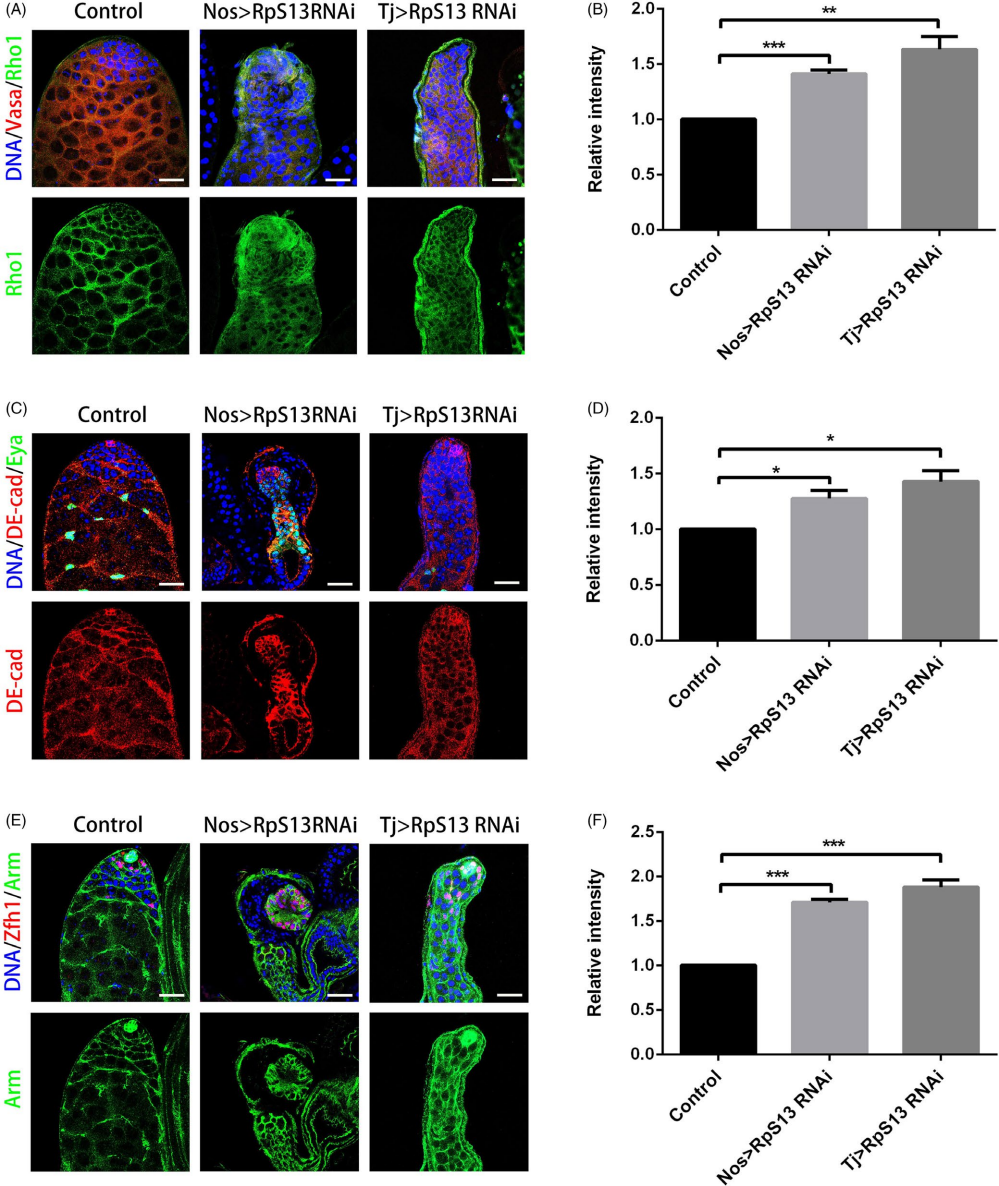
RpS13 regulated Rho1 and cell adhesions in *Drosophila* testes. A, Apical tips of control, nos > RpS13 RNAi and tj > RpS13 RNAi testes labelled with Vasa (red) and Rho1 (green). B, The relative intensity of Rho1 in the control, nos > RpS13 RNAi and tj > RpS13 RNAi testes. C, Apical tips of control, nos > RpS13 RNAi and tj > RpS13 RNAi testes labelled with DE‐cad (red) and Eya (green). D, The relative intensity of DE‐cad in the control, nos > RpS13 RNAi and tj > RpS13 RNAi testes. E, Apical tips of the control, nos > RpS13 RNAi and tj > RpS13 RNAi testes labelled with Zfh1 (red) and Arm (green). F, The relative intensity of Arm in the control, nos > RpS13 RNAi and tj > RpS13 RNAi testes. DNA was stained with Hoechst 33 342 (blue). *P < .05, **P < .01, ***P < .001. Scale bars, 20 µm

**Figure 6:**
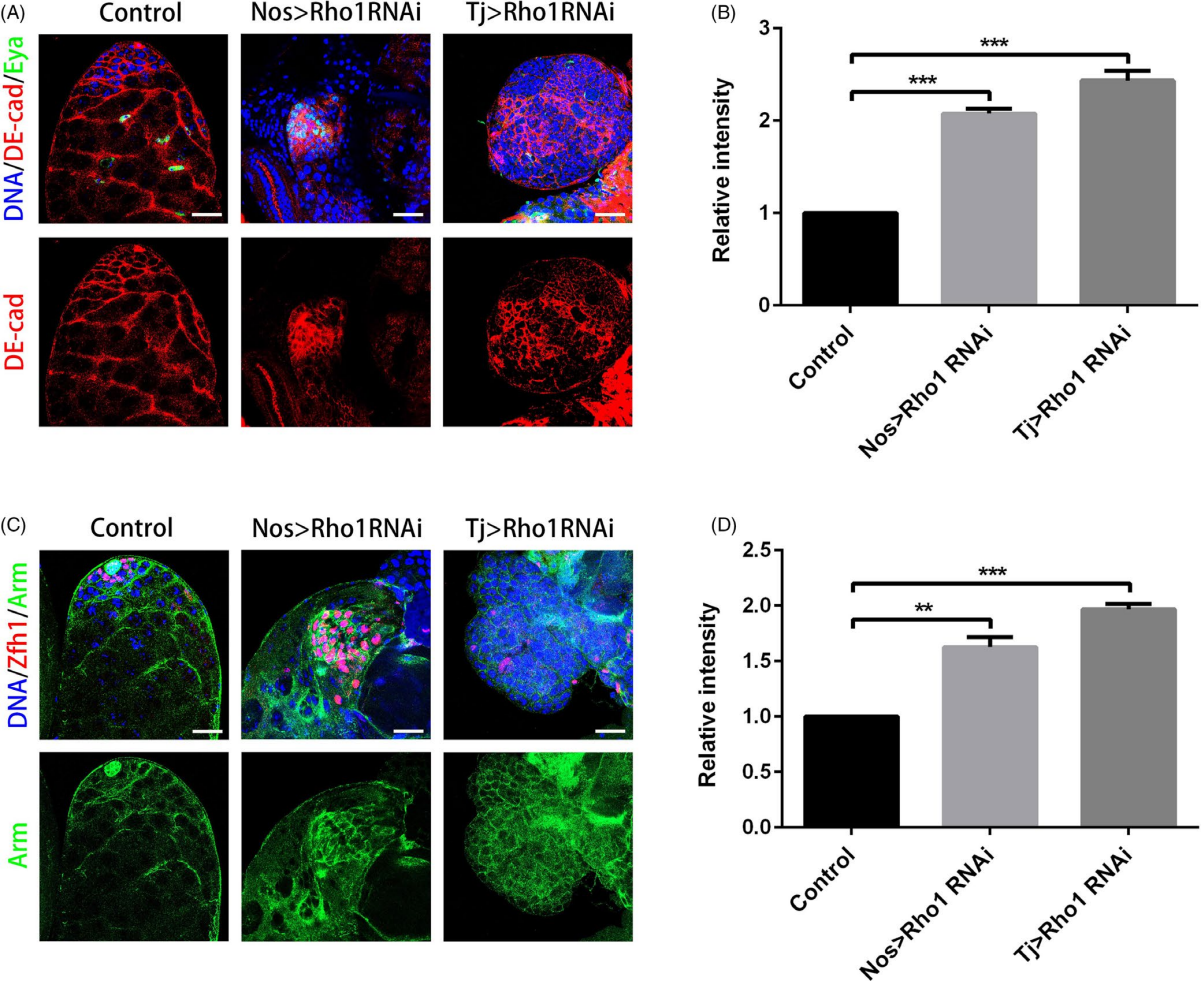
Knockdown of Rho1 led to aberrant cell adhesions in *Drosophila* testes. A, Apical tips of control, nos > Rho1 RNAi and tj > Rho1 RNAi testes labelled with DE‐cad (red) and Eya (green). B, The relative intensity of DE‐cad in the control, nos > Rho1 RNAi and tj > Rho1 RNAi testes. C, Apical tips of control, nos > Rho1 RNAi and tj > Rho1 RNAi testes labelled with Zfh1 (red) and Arm (green). D, The relative intensity of Arm in the control, nos > Rho1 RNAi and tj > Rho1 RNAi testes. DNA was stained with Hoechst 33 342 (blue). **P ＜ .01, ***P ＜ 0.001. Scale bars, 20 µm

To further investigate the relationship between RpS13 and Rho1, we next examined the expression of the Rho1 protein. In controls, Rho1 was highly expressed in somatic cyst cells and could also be detected in germ cells (Figure 5A). Importantly, knockdown of RpS13 in early germ cells and cyst cells led to the accumulation of Rho1 (Figure 5A,B and Figure S3A). We also observed that knockdown of RpS13 both in early germ cells and cyst cells enhanced the accumulation of DE‐cad and Arm (Figure 5C‐F and Figure S3B,C), indicating that RpS13 could moderate cell adhesions in the germline stem cell niche. Moreover, the Rho1 protein level was dramatically decreased in both nos > Rho1 RNAi and tj > Rho1 RNAi testes (Figure S4 and Figure S5A). As expected, DE‐cad and Arm were accumulated in Rho1 RNAi testes driven by both nos‐Gal4 and tj‐Gal4 (Figure 6A‐D and Figure S5B,C). Taken together, these results indicated that RpS13 could regulate the Rho1 expression pattern, moderating DE‐cad‐ and ARM‐mediated cell adhesions in *Drosophila* testes.

### 3.6 RpS13 and Rho1 regulate the expression level of ribosome subunits

**Figure 7:**
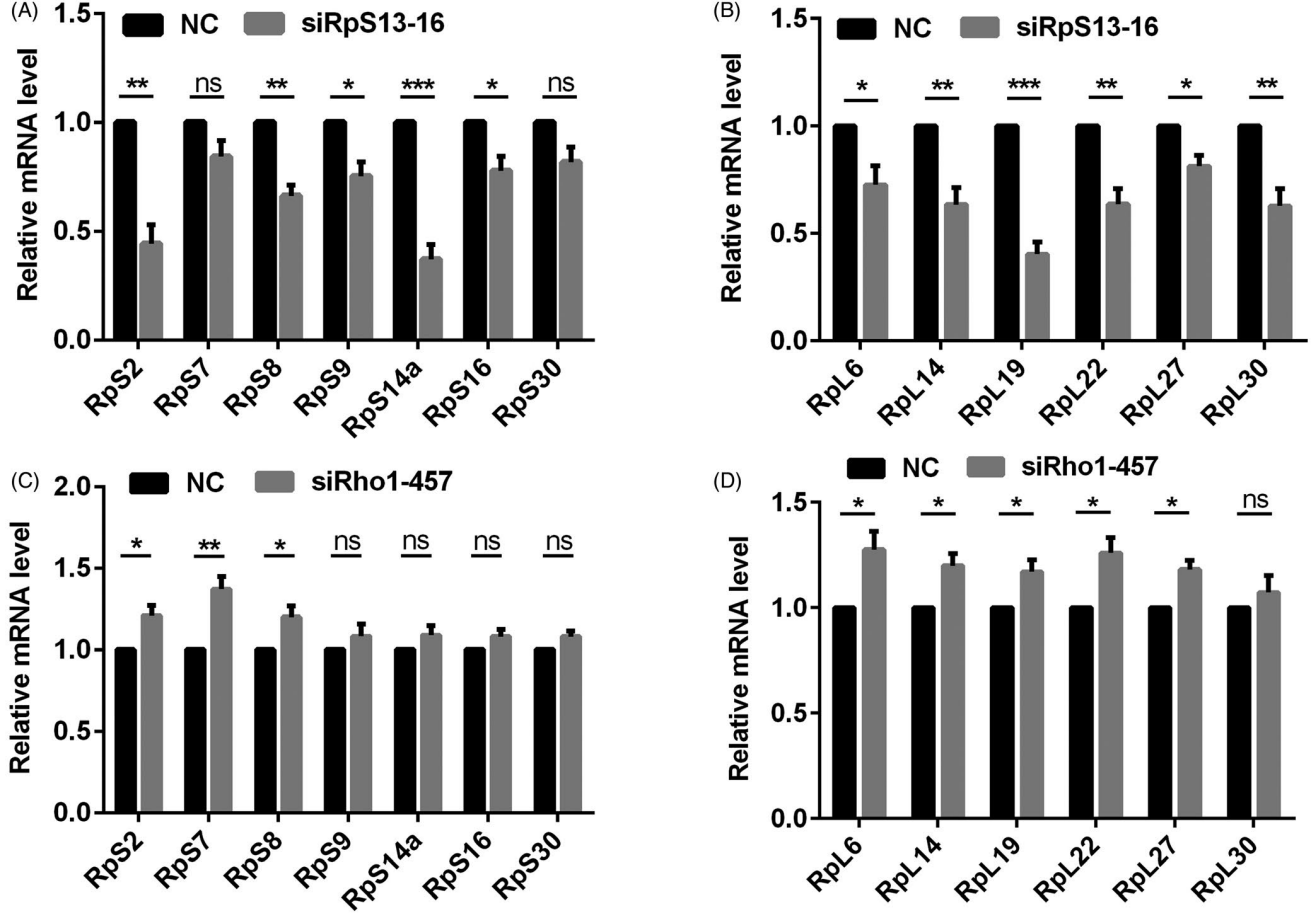
The relative expression level of ribosome subunits. A, Relative mRNA level of small ribosome subunits (RpS2, RpS7, RpS8, RpS9, RpS14a, RpS16 and RpS30) in NC (siRNA) and RpS13 siRNA‐16 S2 cells. B, Relative mRNA level of large ribosome subunits (RpL6, RpL14, RpL19, RpL22, RpL27 and RpL30) in NC (siRNA) and RpS13 siRNA‐16 S2 cells. C, Relative mRNA levels of small ribosome subunits (RpS2, RpS7, RpS8, RpS9, RpS14a, RpS16 and RpS30) in NC (siRNA) and Rho1 siRNA‐457 S2 cells. D, Relative mRNA level of large ribosome subunits (RpL6, RpL14, RpL19, RpL22, RpL27 and RpL30) in NC (siRNA) and Rho1 siRNA‐457 S2 cells. *P ＜ .05, **P ＜ .01, ***P ＜ .001, ns represents no significance

The ribosome is composed of many large and small subunits, which regulate the synthesis of proteins as well as having non‐ribosomal functions. To figure out whether RpS13 and Rho1 affected the functions of the ribosome, we tested the relative mRNA expression level of ribosome subunits in S2 cells. Our results showed that the expression of major small ribosome subunits (eg RpS2, RpS8, RpS9, RpS14a and RpS16) and large ribosome subunits (eg RpL6, RpL14, RpL19, RpL22, RpL27 and RpL30) were downregulated in RpS13 siRNA S2 cells (Figure 7A,B). In contrast, the expression of major small ribosome subunits (eg RpS2, RpS7 and RpS8) and large ribosome subunits (eg RpL6, RpL14, RpL19, RpL22 and RpL27) were upregulated in Rho1 siRNA S2 cells (Figure 7C,D). These results indicated that RpS13 and Rho1 together regulate the functions of the ribosome.

## 4 DISCUSSION

The stability of the niche function is of great importance in regulating stem cell behaviour, replaceability and competition among stem cells.^1^, ^2^, ^3^, ^29^, ^30^ In this study, using *Drosophila* as a model, we identified RpS13 as a crucial factor that could control the homeostasis of GSC fates, demonstrating that RpS13 is essential for GSC self‐renewal and differentiation in the male stem cell niche. Specially, we first provided new insights into the potential regulatory mechanisms between RpS13 and the small GTPase Rho1 signalling protein.

It has confirmed that ribosomal subunits were essential for GSC maintenance and differentiation in both *Drosophila* testis and ovary.^20^, ^21^ And ribosomal subunits could act as an intermediary to affect GSC self‐renewal and differentiation in the *Drosophila* testis.^22^ We showed that both RpS13 and Rho1 could regulate the expression of ribosome subunits. Consequently, it is highly possible that RpS13 and Rho1 affect GSC self‐renewal and differentiation by regulating ribosome functions. These observations emphasized the importance of protein synthesis control in GSC homeostasis and fate decisions. Moreover, Rho1 mimicked the phenotype of RpS13, both in vivo and in vitro. Importantly, our data suggested that RpS13 moderated Rho1, DE‐cad and Arm protein, and Rho1 could further recruit the accumulation of DE‐cad‐ and Arm‐mediated cell adhesions. Therefore, these results indicate that RpS13, together with the small GTPase Rho1, are crucial for GSC self‐renewal and differentiation.

To regulate GSCs, competition between GSCs and CySCs can take place under the control of JAK‐STAT signalling in the male stem cell niche.^4^, ^31^ CySCs drive out GSCs from the stem cell niche and then occupy the site.^32^ The competition between CySCs and GSCs may lead to a reduction in GSCs as a consequence of hyperactivation of Hh signalling in cyst cells.^32^ Accordingly, our results might demonstrate the competition between GSCs and CySCs, resulting in the loss of GSCs and their replacement by CySCs after silencing RpS13 or Rho1 in early germ cells.

Multiple signals are involved in spermatogonial dedifferentiation into germline stem cells to stabilize the stem cell niche.^5^, ^33^, ^34^, ^35^ The process of dedifferentiation is an important factor in the formation of cancer cells. For instance, dedifferentiation behaviour plays roles in the mutation acquisition process of stem cell‐driven cancers, which depend on stem cell homeostasis.^36^ SOX2 imparts stem cell‐like characteristics to human pancreatic cancer cells by promoting dedifferentiation.^37^ Recent studies demonstrated that TGF‐β signalling drives dedifferentiation events to enhance stem cell properties in human colorectal cancer.^38^ Dysfunctional niche signals may lead to a loss of balance between differentiation and proliferation, and contribute to the regulatory mechanisms of cancer in adult stem cell lineages.^39^ CySCs have been proven to regulate the differentiation of GSCs in *Drosophila* testes.^7^ Our study found that knockdown of RpS13 and Rho1 driven by tj‐Gal4 caused GSC differentiation defects, and enhanced the accumulation of dedifferentiation cells. We showed that RpS13 is necessary for the GSC differentiation via the small GTPase Rho1 signals and may prevent the formation of germline lineage induced tumorigenesis.

The Wnt signalling pathway plays diverse roles in various processes, including embryonic development, stem cell maintenance, cell division, adhesion, migration and polarity.^11^, ^40^, ^41^ Several Wnt target genes, including pescadillo ribosomal biogenesis factor 1 (PES1), peter pan homologue (PPAN), block of proliferation 1 (BOP1), nucleophosmin (NPM) and RNA component of mitochondrial RNA processing endoribonuclease (RMRP), have been identified as downstream factors of the Wnt signalling pathway and are involved in mammalian ribosome biogenesis.^42^

Moreover, Wnt ligands have been found to regulate nucleolar size and ribosome composition.^42^ Nucleolar ribosome processing factors, such as eukaryotic translation initiation factor 6 (eIF6), pygopus (Pygo) and thyroid cancer 1 (TC1), participate in the ribosome biogenesis process and regulate Wnt signalling at different levels.^42^ Consequently, the dual role of these factors emphasizes the importance of the connection between ribosomes and Wnt‐related signals for normal cellular functions.

The small GTPases Rho1, Rac1 and Cdc42, as the best‐characterized members of the Rho family of GTPase, have been demonstrated to play a part in the regulation of membrane trafficking, particularly endocytosis. In the *Drosophila* ovary, the simultaneous knockdown of Rho1 in germ cells and escort cells causes cell death and lethality, while the specific knockdown of Rho1 in escort cells results in the loss of germ cells.^43^ Additionally, Rho1 works in escort cells to promote GSCs progeny differentiation by maintaining EGFR signalling and preventing BMP signalling.^44^ The ERK signalling pathway attenuates Rho1 activity to maintain the somatic escort cells’ shape and function.^45^ Rho1, together with Rac1, regulates germ cell enclosure functions via somatic cyst cells in the *Drosophila* testis.^46^ It has been reported that asymmetrically activated Rac1, activated at the niche‐GSC interface along with Cdc42, is necessary for GSC centrosome in localization and is also able to promote BMP signalling in GSCs. ^47^

A deficiency of cell adhesion proteins among niche cells can disintegrate the hub cells’ morphology.^6^ A previous study showed that reduced expression of DE‐cadherin led to GSC loss within the stem cell niche.^48^ In RpS13 RNAi testes driven by nos‐Gal4 and tj‐Gal4, collapse of testicular structures, which were resulted from defects in self‐renewal and differentiation of GSCs, induced the accumulation of Rho1‐, DE‐cad‐ and Arm‐mediated cell adhesions. It has been reported that the deletion of Rho1 resulted in the ectopic accumulation of DE‐cad, independent of Arm.^49^ Our study showed that RpS13 could regulate the Rho1 expression pattern, and RNAi‐mediated silencing of Rho1 led to the enhanced accumulation of DE‐cad‐ and Arm‐mediated cell adhesions in *Drosophila* testes. We also noticed that RpS13 participation in gene regulation and cell adhesions may come in a variety of approaches, and we will continue to explore the possible mechanisms in the future studies.

In conclusion, our biological findings revealed the roles of RpS13 in GSC self‐renewal and differentiation, and identified regulatory mechanisms for the balance of Rho1‐mediated signals in the stem cell niche of *Drosophila* testes. This study uncovered novel clues that link the germline stem cell niche, the ribosome and the small GTPase Rho1 and will be likely to provide new insights for elucidating the pathogenic mechanism of male sterility and testicular germ cell tumours.

## CONFLICT OF INTEREST

The authors declare that they have no conflicts of interest.

## AUTHOR CONTRIBUTIONS

Jun Yu, Bo Zheng and Xia Chen initiated the project and designed the research; Min Wang, Xia Chen, Yibo Wu, Qianwen Zheng and Jie Fang performed most of the experiments, data collection and analysis. Yidan Yan, Wanyin Chen, Xiaojin Luan and Cong Shen assisted in these processes. Min Wang prepared figures under the supervision of Jun Yu and Bo Zheng. Jun Yu wrote the manuscript with the assistance of Min Wang and Qianwen Zheng. Xia Chen, Yibo Wu and Bo Zheng proofread and gave advice. All authors read and approved the final manuscript.

## Supporting information

Supplementary Material

## Data Availability

The data that support the findings of this study are available from the corresponding author upon reasonable request.
