# Supplementary material for: RpS13 controls the homeostasis of germline stem cell niche through Rho1‐mediated signals in the Drosophila testis
Source: Cell Prolif. 2020 Sep 8;53(10):e12899. doi: 10.1111/cpr.12899 (PMC7574871; doi:10.1111/cpr.12899)
Supplement: Supplementary file 1 — Supplementary Material [file CPR-53-e12899-s001.docx]

**Supplementary Information**


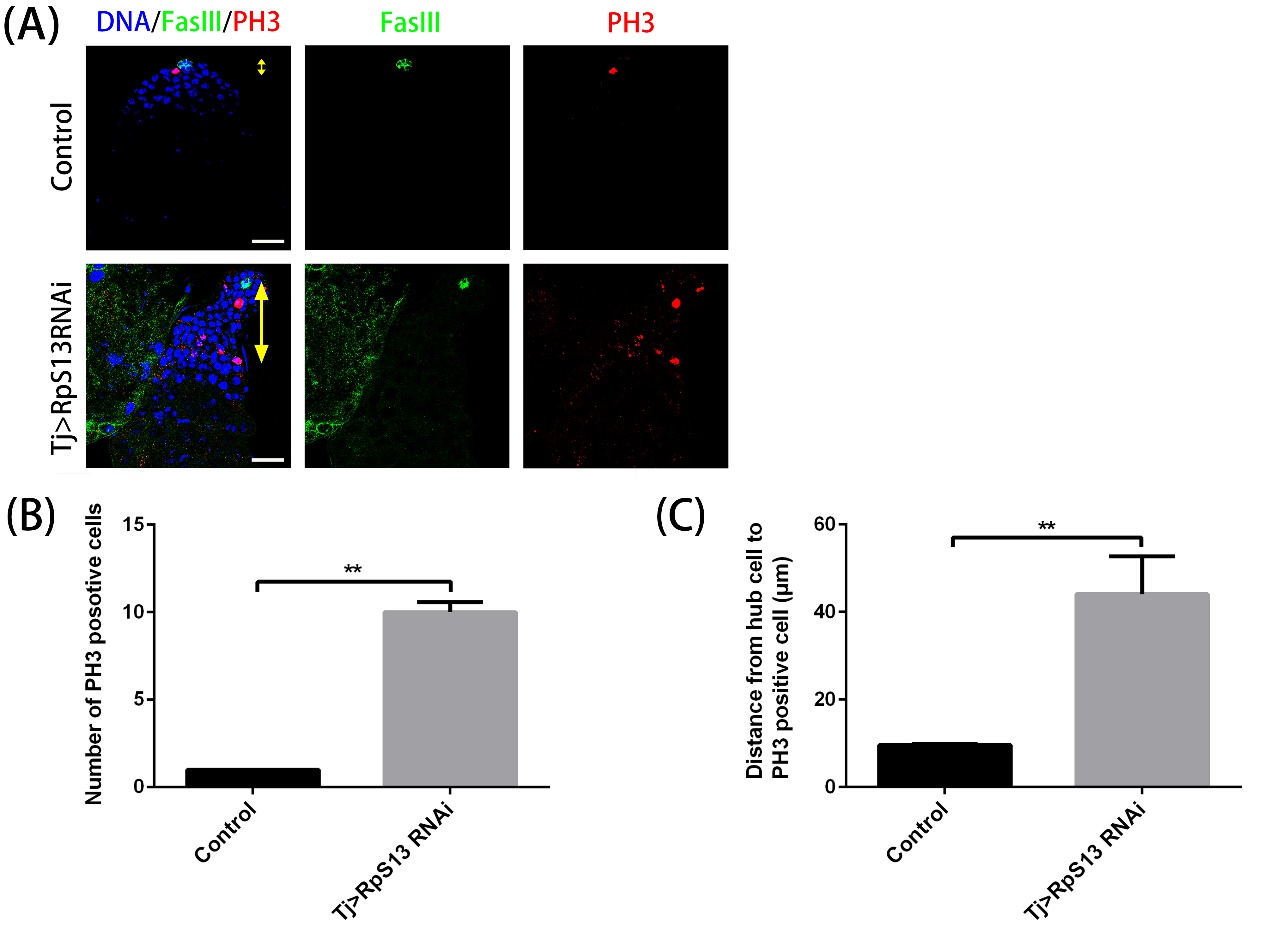


**SUPPLEMENTARY FIGURE 1. Distribution of PH3 staining in RpS13 knockdown testes.** A, Apical tips of control and tj>RpS13 RNAi testes labelled with PH3 (red) and FasIII (green). Yellow two-way arrows indicate the distance between hub cells to PH3 positive cells. B, The number of PH3 positive cells in the control and tj>RpS13 RNAi testes. C, The distance from the hub cell to the PH3 positive cell in the control and tj>RpS13 RNAi testes. DNA was stained with Hoechst 33342 (blue). ***P* < .01. Scale bars, 20 µm.


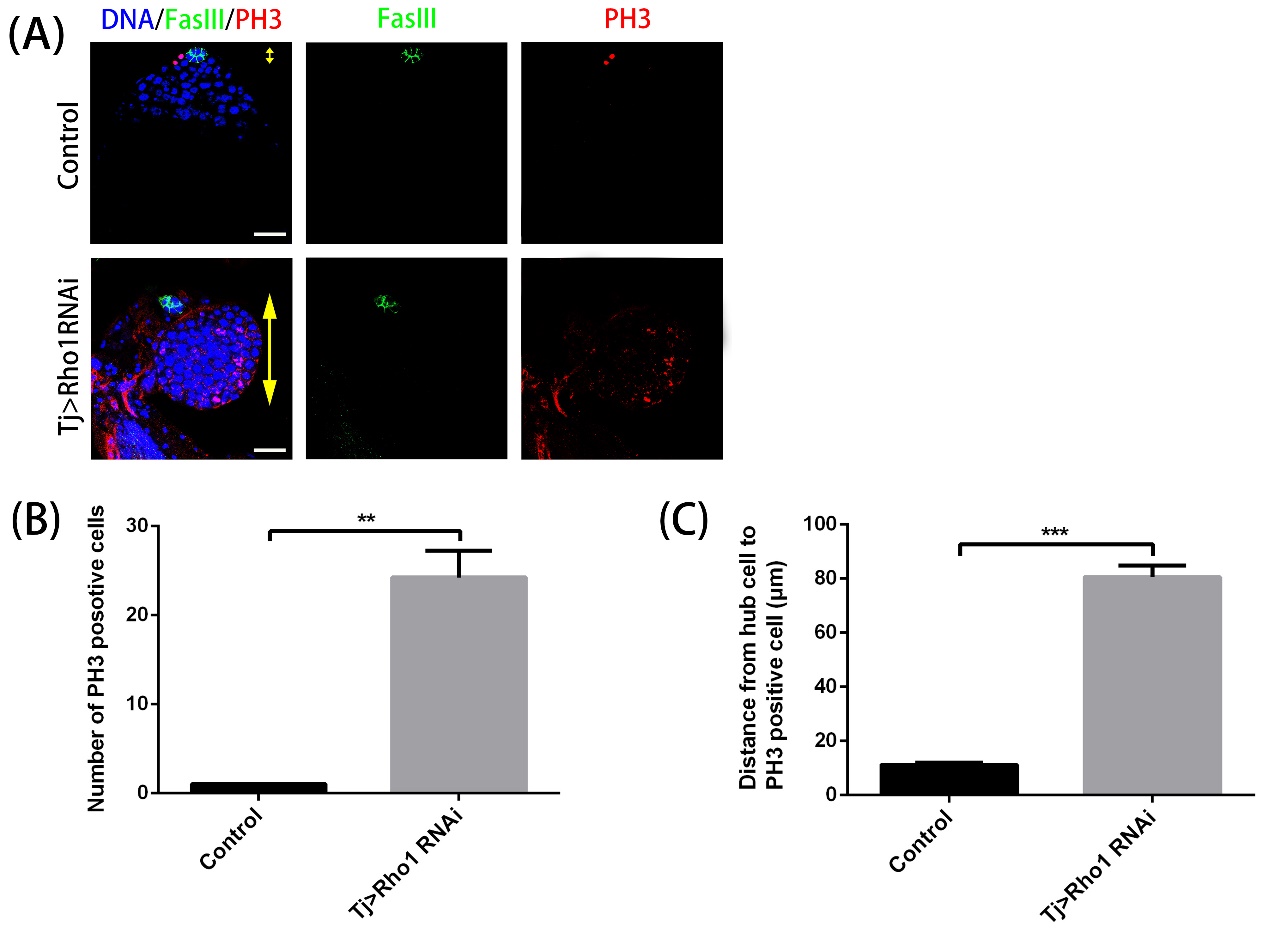


**SUPPLEMENTARY FIGURE 2. Distribution of PH3 staining in Rho1 knockdown testes.** A, Apical tips of control and tj> Rho1 RNAi testes labelled with PH3 (red) and FasIII (green). Yellow two-way arrows indicate the distance between hub cells to PH3 positive cells. B, The number of PH3 positive cells in the control and tj> Rho1 RNAi testes. C, The distance from the hub cell to the PH3 positive cell in the control and tj> Rho1 RNAi testes. DNA was stained with Hoechst 33342 (blue). ***P* < .01, ****P*＜.001. Scale bars, 20 µm.


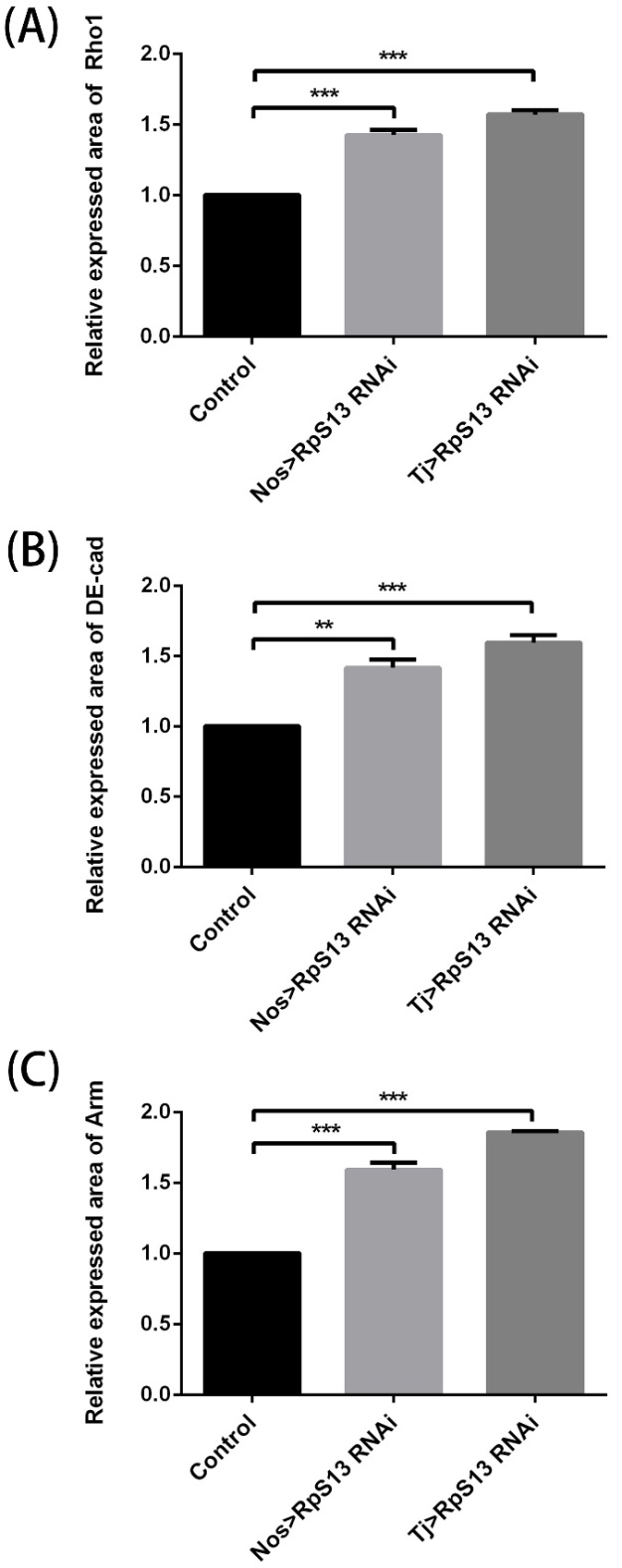


**SUPPLEMENTARY FIGURE 3. The relative expressed area of Rho1, DE-cad and Arm in RpS13 RNAi testes.** A, The relative expressed area of Rho1 in the control, nos> RpS13 RNAi and tj> RpS13 RNAi testes. B, The relative expressed area of DE-cad in the control, nos> RpS13 RNAi and tj> RpS13 RNAi testes. C, The relative expressed area of Arm in the control, nos> RpS13 RNAi and tj> RpS13 RNAi testes. ** *P* < .01, *** *P* < .001.


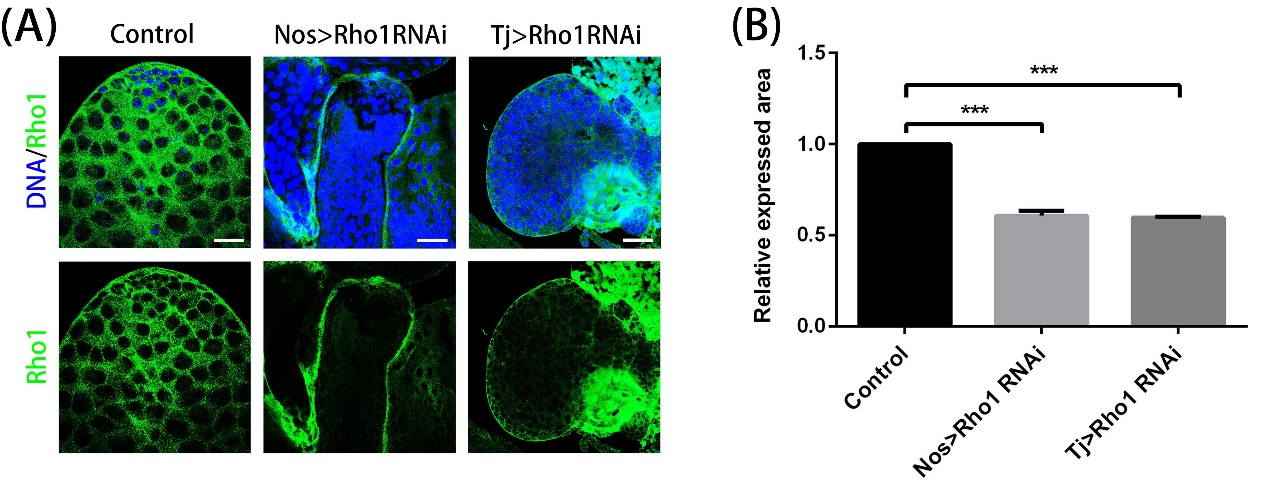


**SUPPLEMENTARY FIGURE 4. Rho1 expression pattern in Rho1 knockdown testes.** A, Apical tips of control, nos>Rho1 RNAi and tj> Rho1 RNAi testes labelled with Rho1 (green). B, The relative intensity of Rho1 in the control, nos> Rho1 RNAi and tj> Rho1 RNAi testes. DNA was stained with Hoechst 33342 (blue). ****P* < .001. Scale bars, 20 µm.


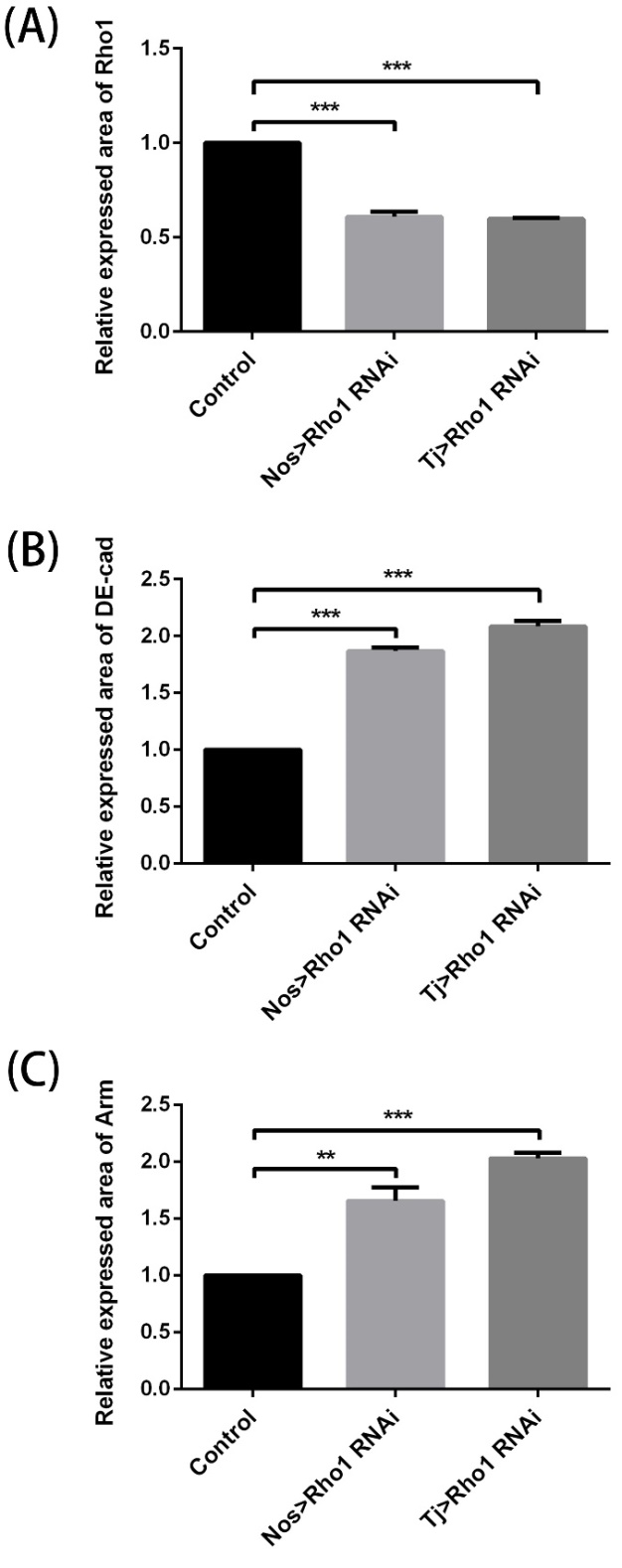


**SUPPLEMENTARY FIGURE 5. The relative expressed area of Rho1, DE-cad and Arm in Rho1 RNAi testes.** A, The relative expressed area of Rho1 in the control, nos> Rho1 RNAi and tj> Rho1 RNAi testes. B, The relative expressed area of DE-cad in the control, nos> Rho1 RNAi and tj> Rho1 RNAi testes. C, The relative expressed area of Arm in the control, nos> Rho1 RNAi and tj> Rho1 RNAi testes. ** *P* < .01, *** *P* < .001.

**SUPPLEMENTARY TABLE 1.** The siRNAs used in this study.

| **Gene** | **Forward (5'-3')** | **Reverse (5'-3')** |
| --- | --- | --- |
| NC | UUCUCCGAACGUGUCACGUTT | ACGUGACACGUUCGGAGAATT |
| RpS13  siRNA-16 | GCUCCUGGCAAGGGUAUUUTT | AAAUACCCUUGCCAGGAGCTT |
| RpS13  siRNA-234 | GGGUCUGAAGCCCGACAUUTT | ACUCCAAAUAGGCAAAGGCTT |
| Rho1  siRNA-457 | GCCUUUGCCUAUUUGGAGUTT | ACUCCAAAUAGGCAAAGGCTT |
| Rho1  siRNA-374 | CCAACACAAUUCGGGAUCUTT | AGAUCCCGAAUUGUGUUGGTT |

**SUPPLEMENTARY TABLE 2.** Primer sequences used in this study.

| **Gene** | **Forward primer (5'-3')** | **Reverse primer (5'-3')** |
| --- | --- | --- |
| GAPDH | GTGGTGAACGGCCAGAAGAT | GCCTTGTCAATGGTGGTGAA |
| RpS2 | GGCCGGTATTGAGGATTGCTACAC | AGGAAGTCCGAGTATGCCTGGTAAG |
| RpS7 | CTGTGTACGACGCCATCCTTGAG | AATGGTGGTCTGCTGGTTCTTGTC |
| RpS8 | TTGGTGAAGAACAGCATCGTGGTC | CTTGGTCAGCACGTCGTTCTCG |
| RpS9 | TCACATTCGTGTCCGCAAGCAG | TCCTCCTCTTCAGCAGCTCCAC |
| RpS13 | CAAGGACAAGGACGGCAAGTTCC | GCAGGACGCTCTTGGTCTTGTAG |
| RpS14a | CTACGCCAGCTTCAACGACACC | GTCTTGCACTTCTCAGCCACATCC |
| RpS16 | AAGGTGAACGGTCGTCCTCTGG | GCTACATGACCACCACCGCTAAC |
| RpS30 | AGCTGTTCGTCCGTGGACTAGAG | AGTTGGTTCTTGACTCCGGCAATG |
| RpL6 | GCACCTGAACGACGCCTACTTC | TCCACCTCCTTCTGGTCCTTCTTG |
| RpL14 | CCTTGCACTCAAGTGAGGAGACAG | GCCAGGCTTGTCGGACATACTTC |
| RpL19 | CCTACCAAGCTGCTGTGGATGC | TGCCTGTCAATCTTCTTGCTGTCG |
| RpL22 | AGGTCAACGGCAAGGTGAACAAC | AAGTGAACGTCGGAGCTGACAATG |
| RpL27 | GGCAAGATCGTAATCGTCCTTAGCG | GGCTTGACCTTGGACTTCTTCTTCAG |
| RpL30 | GTCTGGCGCTGGTGATGAAGTC | CTCGATGTTGGTGCCGCTGTAG |
| Rho1 | GCTGTGGCTGGACGAGATTGTG | TTGACGCCTACGACCTCCTTGG |

**SUPPLEMENTARY TABLE 3.** Primary antibodies used in this study.

| Antigen | Source | Dilution | Company |
| --- | --- | --- | --- |
| Vasa | Rabbit | 1:200 | Santa Cruz Biotechnology |
| 1B1 | Mouse | 1:75 | Developmental Studies Hybridoma Bank |
| Zfh1 | Rat | 1:2000 | A gift from Tong Lab |
| Eya | Mouse | 1:50 | Developmental Studies Hybridoma Bank |
| FasIII | Mouse | 1:50 | Developmental Studies Hybridoma Bank |
| PH3 | Rabbit | 1:400 | Cell Signaling Technology |
| Rho1 | Mouse | 1:50 | Developmental Studies Hybridoma Bank |
| Arm | Mouse | 1:50 | Developmental Studies Hybridoma Bank |
| DE-cad | Rat | 1:20 | Developmental Studies Hybridoma Bank |
